# Thrombocytopenia among pregnant women in Africa: a systematic review and meta-analysis

**DOI:** 10.11604/pamj.2022.41.334.30175

**Published:** 2022-04-26

**Authors:** Solomon Getawa, Zegeye Getaneh, Mulugeta Melku

**Affiliations:** 1 Department of Hematology and Immunohematology, School of Biomedical and Laboratory Sciences, College of Medicine and Health Sciences, University of Gondar, Gondar, Ethiopia

**Keywords:** Thrombocytopenia, pregnant women, meta-analysis, systematic review and Africa

## Abstract

**Introduction:**

thrombocytopenia is a common hematological disorder during pregnancy next to anemia. Pregnant women with thrombocytopenia have complications of excessive bleeding during or after childbirth, cesarean section incision site oozing, stillbirth and neonatal thrombocytopenia. Findings on the magnitude of thrombocytopenia among pregnant women were inconsistent. Therefore, this review aimed to estimate the pooled prevalence of thrombocytopenia among pregnant women in Africa.

**Methods:**

this systematic review and meta-analysis were performed based on PRISMA guidelines. The databases (PubMed, PubMed Central, Hinari, Science Direct, Pop line, Google Scholar, and African Journals Online) were searched to identify relevant studies. Data were analyzed using STATA 11 statistical software. A random-effect model was fitted to estimate the pooled prevalence of thrombocytopenia. I^2^ test statistics were done to test the heterogeneity of included studies. Funnel plots analysis and Egger weighted regression tests were done to detect publication bias.

**Results:**

of the total 1,517 articles retrieved, 15 articles which involved 8,380 pregnant women were eligible for meta-analysis. The overall pooled prevalence of thrombocytopenia among pregnant women in Africa was 10.23% (95% confidence interval (CI): 7.44, 13.02%). Its level of severity showed that, 77.95% (I^2^=43.1%), 15.62% (I^2^=53.4%), and 5.60 (I^2^=0.0%) of pregnant women had mild, moderate and severe thrombocytopenia, respectively. The highest prevalence of thrombocytopenia was occurred in the third trimester of pregnancy (54.05% (95% CI: 29.48, 78.61)).

**Conclusion:**

this systematic review and meta-analysis showed that the pooled prevalence of thrombocytopenia among pregnant women in Africa was found to be relatively higher compared with the globe. Therefore, routine screening and follow-up programs are needed to identify pregnant women with thrombocytopenia and provide them with the necessary interventions.

## Introduction

Thrombocytopenia is a condition in which the number of platelet count is reduced [1]. It is the second leading cause of hematological disorders during pregnancy next to anemia and observed in approximately 6-15% of pregnancies [2,3]. The average number of platelet counts in humans is 150,000-450,000/μL. Thrombocytopenia defined as a platelet count below 150,000 cells/μL and its level of severity classified as mild (100,000-150,000/μL), moderate (50,000-100,000/μL) and severe (less than 50,000/μL) [4]. Studies showed that pregnant women are four times at risk of thrombocytopenia than non-pregnant women [5,6]. During pregnancy, there is usually a decrease in platelet count, mainly due to dilutional effect by increased plasma volume, accelerated platelet destruction, increased platelet aggregation and platelet consumptions in maternal and uteroplacental circulation [7-10].

Gestational thrombocytopenia (GT) is the most common cause of thrombocytopenia during pregnancy [11,12]. It is diagnosed by a reduction in the platelet count in the absence of other clinical and hematological changes [13]. Platelet count in patients with GT is usually >110,000/μL-150,000/μL and responsible for approximately 75% of thrombocytopenia during pregnancy [14]. It is a mild and does not have significant influence on pregnancy, labor, delivery and the neonate [15].

Hypertensive disorders like hemolysis, elevated liver enzymes, and a low platelet (HELLP) syndrome and severe preeclampsia are the second common cause (15-20%) of thrombocytopenia during pregnancy. Hemolytic elevated liver low platelet syndrome causes hemolysis and severe hypertension, alters liver functions, and lowers the platelet count which leads to maternal and fetal morbidity and mortality due to placental abruption, preterm deliveries, intrauterine growth retardation, stillbirths and maternal deaths [16]. Immune thrombocytopenic purpura (ITP) accounts 5% causes of thrombocytopenia during pregnancy. Pregnant women with ITP require treatment due to the risk of maternal hemorrhage and neonatal thrombocytopenia [17]. Infections like malaria, diseases such as aplastic anemia and leukemia and folate deficiency are the causes of thrombocytopenia during pregnancy [18,19].

Thrombocytopenia during pregnancy is underdiagnosed and miss-managed. It has been described in many studies, however, due to the variability of the numbers found in different studies, it is important to carry out more comprehensive analysis such as meta-analysis, to elucidate the real prevalence and variability between different samples. Therefore, this systematic review and meta-analysis was designed to estimate the pooled prevalence of thrombocytopenia among pregnant women in Africa using the available published evidence.

## Methods

**Design and protocol registration:** this systematic review and meta-analysis were designed according to the Preferred Reporting Items for Systematic Review and Meta-Analysis (PRISMA) protocols [20]. The protocol has been registered in the PROSPERO, an International Prospective Register of Systematic Reviews with the registration number of CRD42020171239.

**Search strategy:** data were collected through searching for previously published literature in databases such as PubMed, PubMed Central, Hinari, Science Direct, Pop line, Google Scholar and African Journals Online. The search for the published article is not time-restricted and includes all published articles up to December 31, 2019. The search terms were used separately and in combination using Boolean operators like “OR” or “AND”. Search terms used include “thrombocytopenia”, “hematological profile”, “hematological parameters”, “hematological abnormalities”, “platelet” AND “pregnant women”, “pregnant”, “pregnancy” AND using African search filter (Annex 1) developed by Pienaar *et al*. to identify prevalence studies [21].

**Inclusion and exclusion criteria:** this review include studies that were conducted and published in a peer-reviewed journal; participants were residing in countries belonging to the African continent from all ethnicities, socioeconomic and educational backgrounds. Cross-sectional, case-control and cohort studies which report the outcome of interest were included. Published articles in the English language was eligible for inclusion. Case reports, case reviews and studies conducted among pregnant women but who had comorbidities like; HIV/AIDS and hypertension were excluded from the study.

**Operational definitions of outcomes:** the primary outcome of this systematic review and meta-analysis is to determine the prevalence of thrombocytopenia among pregnant women in Africa. Thrombocytopenia was defined as a platelet count below the established reference interval of the population in the included studies.

**Study selection and quality assessment:** retrieved articles were imported to EndNote X5 (Thomson Reuters, New York, USA) to collect and organize search outcomes and for the removal of duplicate articles. Then, articles were screened by their titles and abstracts by two reviewers (SG and ZG) independently. The disagreement arises between the reviewers was resolved through discussion and the involvement of a third reviewer (MM). Similarly, two reviewers appraised the methodological quality of included studies using the Hoy risk of bias tool [22]. The tool consists of 10 items to assess the internal and external validity such as: 1) representation of the population; 2) sampling frame; 3) methods of participants´ selection; 4) non-response bias; 5) data collection directly from subjects; 6) acceptability of case definition; 7) reliability and validity of study tool; 8) mode of data collection; 9) appropriateness of numerator and denominator; 10) summary of the overall risk. Each item was assessed as either low or high risk of bias. Unclear was regarded as a high risk of bias. Finally, the overall risk of bias was scored according to the number of the high risk of bias per study: low (0-3), moderate (4-6), and high (7-9).

**Data extraction:** relevant studies that fulfilled the eligibility criteria were subjected to data extraction by two authors (SG and ZG) independently and summarized into an excel spreadsheet. Discrepancies were resolved through consensus and discussion with a third author (MM). The following items: authors´ name, year of publication, sample size, total number of cases, age of study participants, study design, thrombocytopenia prevalence with its severity level, the country and the region where the study conducted were extracted for analysis.

**Statistical analysis:** extracted data were entered into Microsoft Excel and then exported to STATA version 11 statistical software for further analysis. Random-effect model meta-analysis was used to estimate pooled effect size and effect of each study with their 95% confidence interval [13]. Forest plots were utilized to estimate the pooled effect size and weight of each recruited study with 95% confidence interval (CI) to show a graphic summary of the data.

**Heterogeneity and publication bias:** the degree of heterogeneity between the included studies was quantified using Higgin´s I^2^ statistics. I^2^ values of 25%, 50%, and 75% are assumed to represent low, medium, and high heterogeneity, respectively [23]. Sub-group analysis by year of publication (2015 and before and after 2015) as well as sensitivity analysis was conducted to determine the potential sources of heterogeneity. Funnel plots analysis and Egger weighted regression tests were done to detect publication bias. P-value < 0.05 in Egger´s test was considered as an evidence of statistically significant publication bias [24,25].

## Results

**Figure 1:**
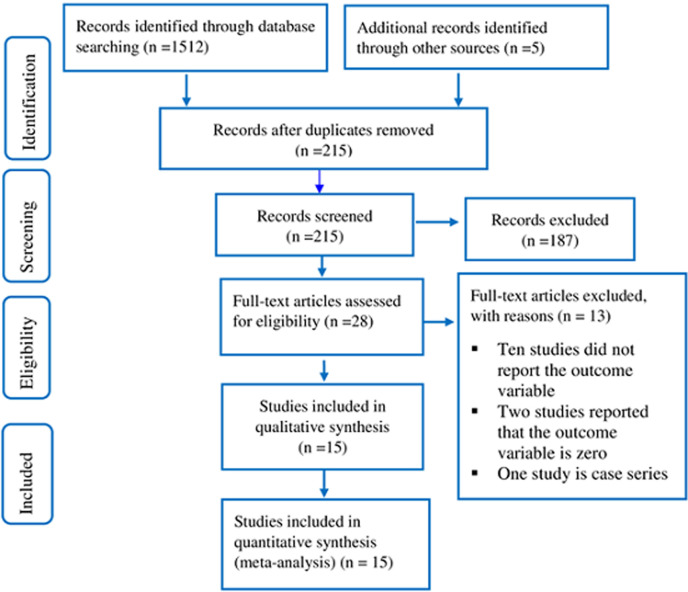
flow chart to describe the selection of studies for the systematic review and meta-analysis

**Literature search and identified studies:** a total of 1,517 studies were identified through database literature searching including manual searching. After removing duplicates, a total of 215 studies were screened for eligibility. Out of which, 187 studies were discarded by reading their titles and 13 studies were removed through reading their full-text. Finally, after excluding non-relevant articles, 15 full-text articles were identified and used for the final qualitative and quantitative analysis (Figure 1).

**Description of included studies:** in this systematic review and meta-analysis, 15 articles were included. Out of 15 articles, five were conducted in Nigeria [26-30], four in Ethiopia [31-34], two in Sudan [17,35] and the remaining were conducted in Ghana [6], Cameroon [36], Libya [37] and South Africa [38]. The sample size of the included study varied from 74 pregnant women in Nigeria [29] to 3520 in Libya [37]. The included studies comprised a total of 8,380 pregnant women, among them 1,060 were thrombocytopenic. Out of 15 included studies, ten studies [6,17,26,27,30-34,37] report the severity of thrombocytopenia as mild, moderate and severe. The methodological quality of the included studies using Hoy risk of bias showed that ten studies (66.67%) have a low risk of bias [6,26,30-34,36-38] and five studies (33.33%) have moderate risk of bias [17,27-29,35]. Moreover, the characteristics of studies included in the meta-analysis were described in (Table 1).

**Table 1:** summary characteristics of included studies in the meta-analysis

Author, year of publication	Country	Study design	Sample size	Cases	Mean age (years)	Prevalence of TCP (%)	Severity of TCP (%)		
							Mild	Moderate	Severe
Shitie *et al*. 2018	Ethiopia	Cross-sectional	284	29	-	10.2	75.86	20.69	3.45
Asrie *et al*. 2017	Ethiopia	Cross-sectional	217	19	25.67±4.69	8.8	74	15.7	10.3
Belaneh *et al*. 2015	Ethiopia	Cross-sectional	193	26	28.9±5.9	13.5	73.07	15.38	11.54
Gebreweld *et al*. 2018	Ethiopia	Cross-sectional	284	22	27.3±4.48	7.7	90.01	9.09	0
Handady *et al*. 2019	Sudan	Cross-sectional	756	79	-	10.4	65.8	7.8	6.4
Mubarak *et al*. 2014	Sudan	Cross-sectional	179	24	26.0±6.8	13.4	-	-	-
Olayemi *et al*. 2012	Ghana	Cross-sectional	300	46	-	15.3	76	20	4
Altayri s, 2017	Libya	Cross-sectional	3520	614	-	17	81	19	0
Erhabor *et al*. 2019	Nigeria	Cross-sectional	120	8	-	6.7	-	-	-
Ajibola *et al*. 2014	Nigeria	Cross-sectional	274	34	-	13.5	78	16	6
Mbanya *et al*. 2007	Cameroon	Cross-sectional	1124	100	25.35±5.48	8.9	-	-	-
Babah *et al*. 2018	Nigeria	Cross-sectional	80	12	31.7±4.16	15.1	91.39	8.61	0
Polycarp *et al*. 2019	Nigeria	Case-control	74	5	28.00±8.29	6.75	-	-	-
Akingbola *et al*. 2006	Nigeria	Cross-sectional	333	12	-	3.6	50	50	0
Sebitloane, 2016	South Africa	Cross-sectional	642	30	-	4.7	-	-	-

**Note**: TCP= Thrombocytopenia

**Figure 2:**
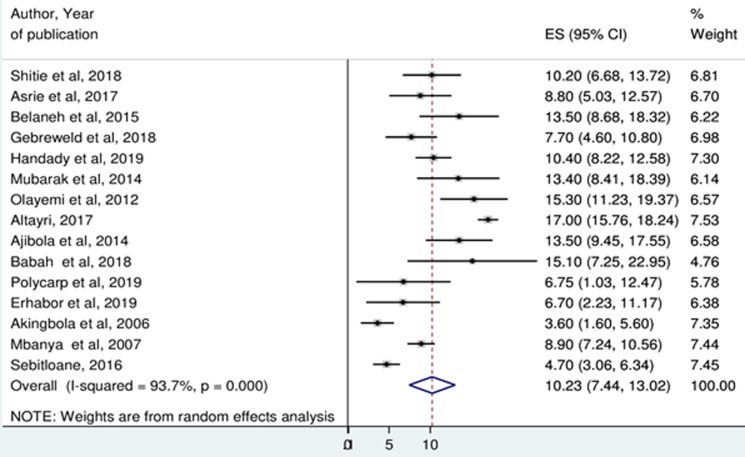
forest plot showing the pooled prevalence of thrombocytopenia among pregnant women in Africa

**Figure 3:**
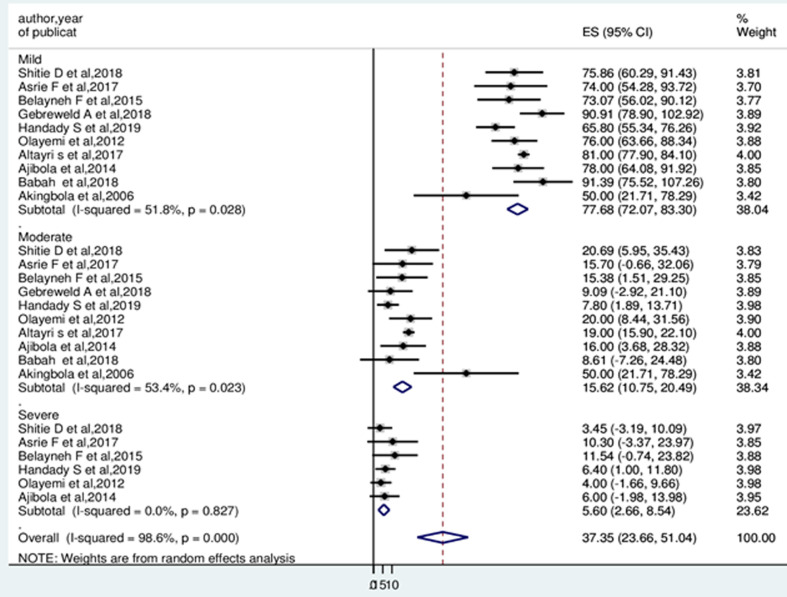
forest plot displaying the severity of thrombocytopenia among pregnant women in Africa

**Prevalence of thrombocytopenia:** the minimum prevalence of thrombocytopenia was 3.6% in Nigeria [27] and the maximum was 17% in Libya [37]. Random-effects model analysis showed that, the pooled prevalence of thrombocytopenia among pregnant women in Africa was 10.23% (95%CI: 7.44, 13.02%). The Higgin´s I^2^ statistics test showed a high heterogeneity between included studies (93.7%, p ≤ 0.001) (Figure 2). Meta-analysis of ten articles on severity of thrombocytopenia revealed that, among thrombocytopenic pregnant women, 77.68% (I^2^=51.8%), 15.62% (I^2^=53.4%), and 5.60 (I^2^=0.0%) had mild, moderate and severe thrombocytopenia, respectively (Figure 3).

**Figure 4:**
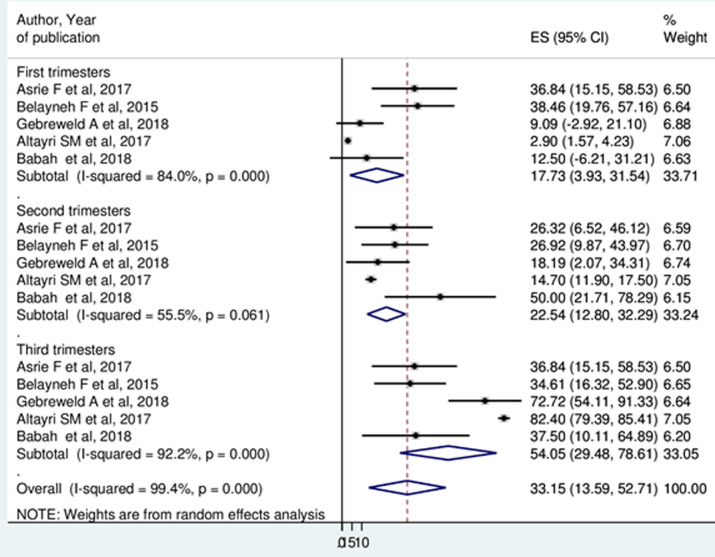
forest plot displaying prevalence of thrombocytopenia among pregnant women by trimesters

**Prevalence of thrombocytopenia in trimester:** five studies [28,31,32,34,37] were report the prevalence of thrombocytopenia in each trimesters of pregnancy. The result showed that, 17.73% (95%CI: 3.93, 31.54) and 22.54% (95%CI: 12.80, 32.29) of the pregnant women were thrombocytopenic in the first and in the second trimesters of pregnancy. In the third trimesters of pregnancy, 54.05% (95%CI: 29.48, 78.61) of pregnant women were thrombocytopenic (Figure 4).

**Figure 5:**
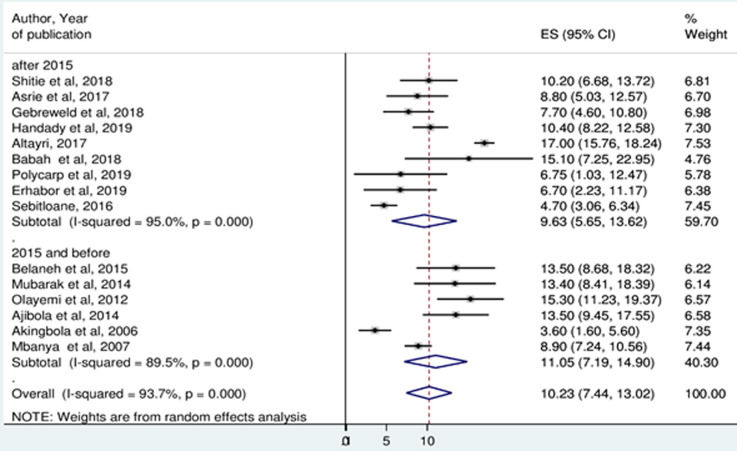
pooled estimates of the prevalence of thrombocytopenia among pregnant women by year of publication

**Figure 6:**
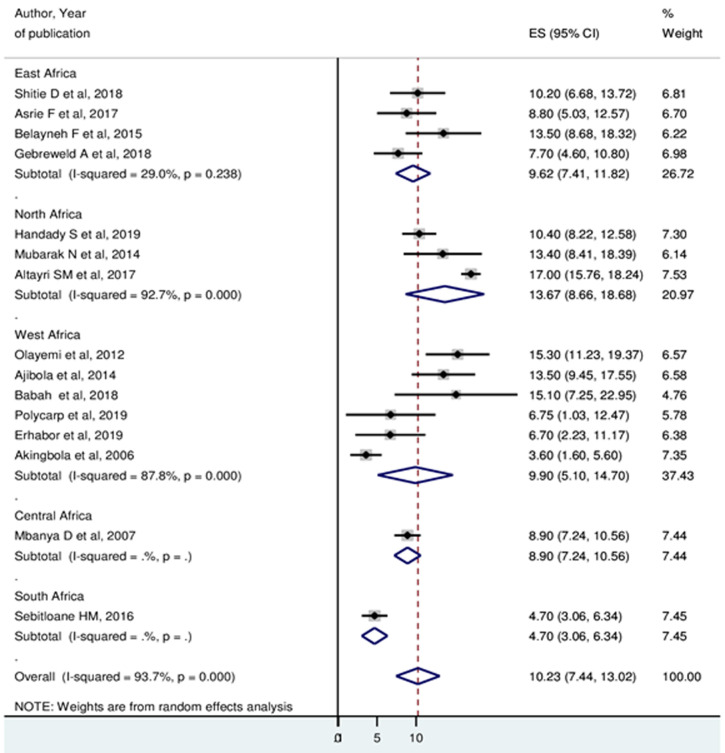
the pooled estimates of the prevalence of thrombocytopenia among pregnant women by Region

**Subgroup analysis:** subgroup analysis on year of publication showed that, the prevalence of thrombocytopenia among studies published 2015 and before was 11.05% (95%CI: 7.19, 14.90%), while for studies published after 2015 was 9.93% (95%CI: 5.65, 13.62%) (Figure 5). Based on the Region, the highest prevalence of thrombocytopenia was observed in the North African region 13.67% (95%CI: 8.66, 18.68%) whereas the lowest prevalence was in South African region 4.7% (95%CI: 3.06, 6.34%) (Figure 6).

**Figure 7:**
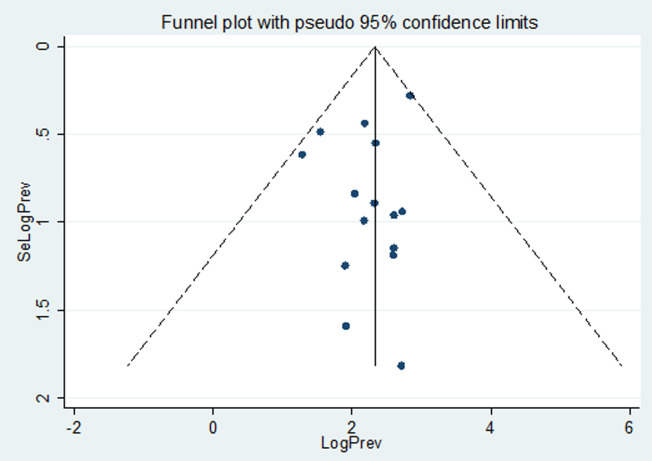
funnel plot of included studies on the prevalence of thrombocytopenia among pregnant women

**Publication bias:** potential publication bias was assessed visually by funnel plot. The funnel plot of the included studies is symmetric and it seems that the publication bias would not be indicative since greater than 100% of the studies fell within the triangular region (Figure 7). Besides, the Egger´s weighted regression statistics indicated that there is no publication bias (P=0.699) (Table 2).

**Table 2:** Egger's test

Std Eff	Coef.	Std. Err.	T	P>t	95%CI	
Slope	11.39754	2.841571	4.01	0.001	5.258701	17.53638
Bias	-0.879422	2.228149	-0.39	0.699	-5.693044	3.9342

**Sensitivity analysis:** due to the high heterogeneity of results, a sensitivity analysis was done by omitting each study step by step to assess the effect of each study on the pooled estimated prevalence. The result showed that omitted studies did not show a significant effect on the pooled prevalence of thrombocytopenia among pregnant women (Table 3).

**Table 3:** sensitivity analysis of the included studies to estimate the pooled prevalence of thrombocytopenia among pregnant women

Study omitted	Estimate(95%CI)	Heterogeneity	
		I2	P-value
Shitie *et al*. 2018	10.24(7.29, 13.19)	94.0%	<0.001
Asrie *et al*. 2017	10.34(7.4, 13.28)	94.0%	<0.001
Belaneh *et al*. 2015	10.02(7.11, 12.92)	93.9%	<0.001
Gebreweld *et al*. 2018	10.42(7.47, 13.38)	94.1%	<0.001
Handady *et al*. 2019	10.23(7.18, 13.28)	94.2%	<0.001
Mubarak *et al*. 2014	10.03(7.12, 12.93)	94.1%	<0.001
Olayemi *et al*. 2012	9.88(6.98, 12.77)	94.0%	<0.001
Altayri, 2017	9.45(7.48, 11.45)	82.5%	<0.001
Ajibola *et al*. 2014	10.00(7.08, 12.92)	94.1%	<0.001
Babah *et al*. 2018	9.99(7.12, 12.85)	94.1%	<0.001
Polycarp *et al*. 2019	10.45(7.56, 13.34)	94.1%	<0.001
Erhabor *et al*. 2019	10.47(7.57, 13.38)	94.1%	<0.001
Akingbola *et al*. 2006	10.74(8.01, 13.49)	92.5%	<0.001
Mbanya *et al*. 2007	10.36(7.22, 13.49)	94.1%	<0.001
Sebitloane, 2016	10.67(7.90, 13.43)	92.2%	<0.001
Combined	10.23(7.44, 13.44)	97.3%	<0.001

**Note:** CI=confidence interval

## Discussion

This systematic review and meta-analysis were carried out to determine the pooled prevalence of thrombocytopenia among pregnant women in Africa by including 15 studies. The pooled prevalence of thrombocytopenia among pregnant women in Africa was 10.23% (95% CI: 7.44, 13.02%). The result of this meta-analysis was comparable to Myers literature review (8%-10%) [39], Jodkowska *et al*. (7%-10%) [19] and Boehlen *et al*. (6%-15%) [3]. However, the result was higher than the recent systematic review and meta-analysis 8.4% (95% CI: 6.9, 10.1%) [11] in the globe. The possible explanation for the difference could be attributed to variations in study design, sample size and difference in cutoff points to define thrombocytopenia. Moreover, a possible reason for the increment of thrombocytopenia in this study might be related to variations in local nutritional status and prevalence of intestinal parasitic infection during pregnancy. On the contrary, the results was lower than individual studies that were conducted in Libya 17% [37], Ghana 15.3% [6], and Nigeria 15.1% [30]. The difference might be related to asymptomatic malaria cases as well as intestinal parasitic infection in the study participants that may aggravate thrombocytopenia [6].

Based on levels of severity, 77.68% (95%CI: 72.07, 83.30%) of pregnant women were mild type thrombocytopenia. This could be caused by GT due to hemodilution, an increment of platelet size, platelet activation, and increased platelet clearance. This is usually not associated with adverse effects for the mother and infant [40]. On the contrary, 5.60% (95%CI: 2.66, 8.54) of pregnant women were severely thrombocytopenic. Severe thrombocytopenia during pregnancy associated with increases the risk of bleeding during cesarean section, postpartum hemorrhage (PPH), neonatal asphyxia, and neonatal thrombocytopenia [2,41].

The highest prevalence of thrombocytopenia occurred in the third trimester of pregnancy 54.05% (95%CI: 29.48, 78.61). The finding was in line with previous studies conducted in the globe [6,32,42,43]. The possible explanation could be an increase in platelet aggregation especially during last 8 weeks of gestation. It has been reported that significant fall in platelet count can occur from 32 weeks of gestation onwards due to hemodilution, increased platelet activation and consumption [44,45]. Subgroup analysis by year of publication showed that 11.05% (95% CI: 7.19, 14.90) and 9.93% (95% CI: 5.65, 13.62) of studies published 2015 and before and studies published after 2015 were thrombocytopenic respectively. This may indicate that an improvement in the health care services of antenatal care or nutritional status of pregnant women through time.

In this study the heterogeneity of the included study was high (I^2^= 93.7%). The possible reason might be related to variations in sample size, study design, and case definition. Inclusion of studies published only in English language that may compromise representativeness and may cause heterogeneity. Besides, in this meta-analysis the publication bias was not observed, Egger´s test with the p-value greater than 0.005 (p=0.699). In the current review thrombocytopenia is a common hematological disorder during pregnancies in those of the included studies. Different factors such as anemia, preeclampsia, hypertensive disorders, malaria, and HIV infection may affect thrombocytopenia among pregnant women [34,36,37].

**Limitation and strength:** the limitation of the study is that the analyzed pooled prevalence lacks representativeness because only articles from seven countries from the African Region were included. On one hand, comprehensive search using different database and different searching strategy was the strength of this study.

## Conclusion

This systematic review and meta-analysis showed that the pooled prevalence of thrombocytopenia among pregnant women in Africa was found to be relatively higher compared with global prevalence. Therefore, adequate intervention should be designed and routine screening and follow-up programs are needed to identify pregnant women with thrombocytopenia, for early detection and treatment of possible complications in order to reduce maternal and neonatal morbidities.

### What is known about this topic

- Thrombocytopenia is second leading cause of hematological disorders after anemia during pregnancy;
- Thrombocytopenia is observed in approximately 6-15% of pregnancies;
- Gestational thrombocytopenia is the main cause thrombocytopenia during pregnancy.

### What this study adds

- The prevalence of thrombocytopenia among pregnant was found to be 10.23% (nearly 10 cases per 100 pregnancies) in Africa;
- Thrombocytopenia among pregnant women in Africa is relatively higher compared with the globe;
- The highest prevalence of thrombocytopenia is occurred in the third trimester of pregnancy.

## Annex

**Annex 1**: search strategy for PubMed (PDF-407 KB)
